# Green credit, environmental protection investment and debt financing for heavily polluting enterprises

**DOI:** 10.1371/journal.pone.0261311

**Published:** 2021-12-15

**Authors:** Li Ji, Pan Jia, Jingshi Yan

**Affiliations:** School of Accounting, Southwestern University of Finance and Economics, Chengdu, Sichuan, China; Universiti Malaysia Sabah, MALAYSIA

## Abstract

The paper takes listed companies in the heavily polluting industry from 2009–2017 as a research sample to explore whether heavy pollution enterprises’ environmental protection investment helps their debt financing under the institutional background of China’s continuous implementation of green credit policy. It is found that, in general, the environmental protection investment of heavy pollution enterprises helps them to obtain more and relatively long-term new loans; in terms of time, this effect is more evident after the release of China’s Green Credit Guidelines in 2012; in addition, the level of regional environmental pollution, the level of financial development and the green fiscal policy also have a moderating effect on this. This paper enriches the study of the economic consequences of corporate environmental protection investment from the perspective of debt financing. It examines the effects of the implementation of China’s green credit policy and other institutional factors to provide a reference for the heavy pollution enterprises’ environmental protection investment and the implementation of green credit policy by local governments in China.

## Introduction

China’s economy has experienced nearly four decades of rapid growth. However, during the process, problems such as tightened resource constraints, severe environmental pollution, and degraded ecosystems have appeared, driving the capacity of both resources and the ecological environment to the limit. The country will face an increasingly stringent resource constraint and enormous costs for ecological restoration in the future. In 2016, for the first time, the 13th Five-Year Plan put the "Green" development together with "innovative, coordinated, open and shared development" as the new strategy, direction, and focus of China’s development. The idea is expected to guide various fields and stages of economic and social development. According to the green development target proposed in the 13th Five-Year Plan for 2016–2020, the annual investment demand for China’s green industry is about 3 trillion yuan. According to the UN report "Establishing China’s Green Financial System", it is estimated that the government’s financial resources can only meet 10%-15% of the green investment needs, and the market needs to play its role in resource allocation for the rest. Green finance became a policy tool for macroeconomic regulation, which mainly targets scarce natural and environmental resources and can help the country effectively allocate resources nationwide, thus promoting stable economic growth. Green credit is the earliest, largest, and most mature part of China’s green financial system. In recent years, the scale of China’s green credit market has grown steadily, with the credit scale of 21 major banks increasing from 4.85 trillion yuan at the end of June 2013 to 8.22 trillion yuan at the end of June 2017, an increase of 69.5%.

Green credit originated in western developed countries, and in 2002, the International Finance Corporation and ABN AMRO Bank pioneered the "Equator Principles," which require financial institutions to fully consider the environmental and social impacts of investment projects when placing credit funds. Subsequently, academics gradually paid attention to the topic of green credit. As an innovative financial instrument, green credit is considered a bridge between the economy and the environment [1]. In the early research stage, scholars mainly discussed the existing challenges and development points of green credit. Aizawa and Yang [2] argue that if a bank lends money to investment projects with negative environmental consequences, the bank’s reputation will also be affected by the negative environmental consequences. The equatorial principle will help solve this problem and level the playing field. Duan and Niu [3]suggest that the rule of law environment is an institutional guarantee for green financial services to the real economy.

With the development of green finance internationally, China has also started to vigorously promote it, among which green credit is one of the earliest and fastest-growing green financial instruments in China. Since 2012, when China released its green credit guidelines, the document has extensively promoted the development of green credit. However, research on green credit policies in China is still in its infancy. Early studies were mainly macro-level studies, landing mainly on China’s industrial structure and economic development [4–6]. In contrast, green credit involves more subjects, including environmental protection departments representing the central government, local governments, banks and enterprises, and other stakeholders. Therefore, the subsequent studies have been intensified, and scholars have started to explore the role of policies for micro subjects. Firstly, it is for banks, the executors of green credit policy. After the national implementation of the green credit policy, banks consider the environmental impact of enterprise products and their sustainable development when granting loans to enterprises [7]. However, banks are more concerned with short-term profits. When there are loopholes in external regulation, banks are likely to disregard environmental protection and other related regulations and put too low a standard on environmental protection for enterprises, so that the loan threshold for heavily polluting enterprises becomes low [8]. However, compared to studies based on commercial banks’ perspectives, scholars have less research on green credit policies at the firm level. The essence of green credit policy is environmental policy, so for firms, the main literature studies revolve around verifying whether the Porter effect holds [9–12].

In summary, most of the current studies on green credit policies in China focus on assessing the effectiveness of green credit policies in different regions from a macro level or the impact of green credit policies on banks’ profitability from the banks’ perspective. In contrast, relatively few studies focus on enterprises as the focus of the policy. First of all, all the literature is generalized with all listed companies as the sample. Since green credit policy is essentially an environmental policy, it is more evident for enterprises with serious pollution emissions, so this paper refines the research sample and conducts an in-depth discussion around heavily polluting enterprises. Second, China has introduced a considerable number of policies on environmental issues in recent years, and the green credit policy is also used as one of the national macro-control instruments. Under several strong state environmental regulations, Chinese companies themselves take many environmental protection, improvement, and transformation actions. One of the most important initiatives is the significant increase in environmental investment. The increase in corporate environmental investment meets policy requirements and enhances its image and public demand, enhancing its reputation, bringing many subsequent benefits, which is an essential improvement in financing convenience. Then this paper clarifies the relationship between heavy pollution enterprises’ environmental investments and debt financing before is a prerequisite for further research on the micro governance role of green credit policy. Finally, the green credit policy, at the same time as a financial instrument, can be through the market-based means of effective macro regulation of funds. Then it will inevitably affect the investment and financing behavior of an enterprise in the first place. Therefore, this paper attempts to take enterprises’ investment and financing behavior as the entry point while studying the micro governance role of green credit policy on heavily polluting enterprises.

This paper attempts to address the following questions: 1. How do the heavy pollution enterprises, most affected by environmental policies, affect their environmental investment in debt financing? 2. What are the strategic changes that firms make in the face of China’s newest and fastest-growing environmental policy, green credit? To address the above questions, this paper selects A-share listed companies in heavy pollution industries in China’s Shanghai and Shenzhen markets from 2009–2017 as a sample to clarify the relationship between firms’ environmental investment and debt financing. It also explores whether it can provide financing convenience for heavy polluting enterprises in the institutional context of China’s continuous implementation of green credit policy.

The research of this paper originates from the concern and reflection on the rapidly developing green finance and the shortage of theories and literature on green credit research in the past. Hence, the innovation of this study is that: In terms of literature, this paper enriches the research on the economic consequences of enterprises’ environmental protection investment from the perspective of debt financing. It examines the effects of green credit policies on firms in China and other institutional environmental factors and expands the research on green credit related to micro firms. Specifically, it enriches and deepens the research on enterprises in unique heavy pollution industries. In terms of practice, although countries have recognized the importance of green credit development, most of them are at the stage of qualitative green credit and green credit evaluation system construction and lack quantitative research on green credit. The research in this paper has some reference value for the subsequent policy implementation and local government implementation of green credit policy in China.

The rest of this paper is organized as follows: the second part presents the research hypothesis of this paper based on the institutional background and theoretical analysis of green credit in China; the third part is the research design; the fourth part is the empirical results and analysis; the fifth part is the further analysis of the mechanism of action; and finally, the research conclusion and indications.

## Institutional background, theoretical analysis and research hypothesis

### Background of the green credit policy

As the earliest green financial instrument in China’s green financial system, green credit had taken shape as early as 1995, when the People’s Bank of China issued the Notice on Implementing Credit Policy and Strengthening Environmental Protection. It offered provisions for the financial sector to implement the national environmental policy in credit-related work, requiring financial departments at all levels to protect natural resources and the environment in credit-related work and to put supporting the ecological resources protection and pollution prevention and control as one of the considerations in issuing bank credit. Further, in 2007, the former State Environmental Protection Administration, the People’s Bank of China, and the China Banking Regulatory Commission (CBRC) have jointly issued the "Opinions on Implementing Environmental Protection Policies and Regulations to Prevent Credit Risks", which more clearly states that environmental supervision and credit management of enterprises and construction projects have become an urgent task. In 2012, the CBRC issued the "Green Credit Guidelines", which expressly set out the requirements for the development of green credit by banking institutions and is a programmatic document for the development of green credit business by financial institutions in China. The Green Credit Guidelines, which elevated green credit to an unprecedented strategic height for the first time, not only had a guiding value but also had practical significance, marking the stage of comprehensive development of China’s green credit policy. Subsequently, the "Green Credit Statistical System" was set up in 2013, and the "Comprehensive Assessment and Evaluation System for the Implementation of Green Credit" was implemented in 2015. With these measures in place, China has constantly been regulating and promoting the development of green credit.

As shown in Table 1, the green credit balance of the five major banks in China is growing year by year. Among them, the Industrial and Commercial Bank of China (ICBC)’s green credit was the largest in 2009, and its pace of development in recent years is also in the leading position among the five major banks. China Construction Bank (CCB) has developed rapidly after the promulgation of the Green Credit Guidelines, with the fastest growth rate, and its scale of green credit became the second largest among the five major banks in 2017. The scale of green credit of the other five major banks has been growing steadily. In general, since the idea of green credit has been proposed, the scale of green credit of the five largest banks in China has been growing year by year and at a faster rate, and the overall participation is high.

**Table 1:** Green credit balance of China’s top five banks from 2009–2017 (billion yuan).

Bankname	2009	2010	2011	2012	2013	2014	2015	2016	2017
ICBC	4080.00	5074.52	5904.00	5934.00	6552.81	8117.47	9146.03	9785.60	10991.99
CCB	1811.00	1958.06	2190.70	2396.37	4883.90	4870.77	7335.63	8892.21	10025.21
BCM	578.00	1022.93	1235.36	1440.28	1658.36	1524.31	2047.95	2411.99	2771.08
ABC	511.00	597.13	881.68	1522.00	3304.21	4724.47	5431.31	6494.32	7476.25
BOC	1503.00	1921.12	2494.00	2274.80	2587.59	3010.43	4123.00	4673.00	5387.99

Data source: Wind.

**Fig 1:**
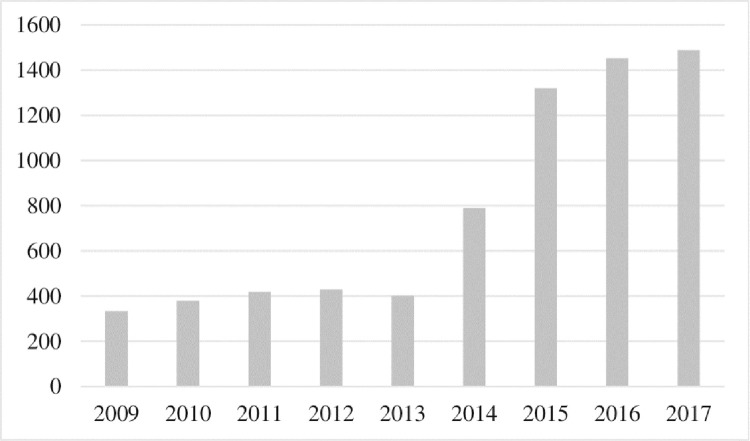
Changes in the scale of environmental protection investment in heavy polluting enterprises from 2009–2017 (billion yuan).

In the face of the increasing role of financial institutions in environmental governance, green credit policies also significantly impact enterprises making environmental protection investment decisions. Fig 1 shows the data of total annual environmental protection investment of heavy pollution enterprises from 2009–2017, which provides a preliminary description of the changes in the scale of environmental protection investment of heavy pollution enterprises before and after the implementation of the green credit policy. From the Fig 1, we can find that:(1) before 2012, the scale of environmental protection investment of heavily polluting enterprises did not change much. (2) After 2012, since the green credit policy was formally introduced in February, it took some time for the market to react and the credit review. Therefore, from 2013, there has been a significant increase in the scale of environmental protection investment of heavy pollution enterprises at an inflection point. This indicates that the scale of environmental protection investment of heavily polluting enterprises has been significantly increased due to the financial support from the green credit policy.

### Environmental protection investment and debt financing for heavy pollution enterprises

Richardson et al. [13] found that social responsibility information disclosed by companies has three mechanisms of effect on themselves: influencing stakeholders’ risk predictions, alleviating information asymmetry and thus reducing transaction costs, and catering to investors’ social responsibility preferences. As part of social responsibility information, environmental information plays an equally important role in the capital market. Clarksori et al. [14] find that a firm’s performance in terms of environmental responsibility affects its debt financing activities and serves as an essential basis for creditors to predict its future operating conditions and evaluate credit risk. Ye, Chen-Gang et al. [15] found that higher quality of corporate environmental information disclosure is negatively related to the cost of equity financing. Wu, Hongjun et al. [16] found that enterprises can significantly reduce financing constraints by improving the level of environmental information disclosure. In summary, it can be seen that by improving the level of environmental information disclosure, enterprises can show good environmental risk management capabilities and corporate development prospects, which can effectively enhance investor confidence and thus reduce the level of financing constraints.

As the public’s awareness of environmental protection increases, corporate stakeholders are becoming increasingly concerned about the negative impact of environmental issues on corporate production and operation. Banks and other creditors also gradually realize that environmental pollution problems may cause credit risk and introduce corporate environmental performance into credit risk assessment. Based on signaling theory, enterprises with better environmental performance tend to have more incentive to disclose their environmental performance to send green signals to stakeholders and establish an excellent environmental image. Enterprises usually tend to disclose favorable environmental information when they choose their disclosure content to demonstrate their environmental responsibility and sustainable development, prompting banks to reasonably assess the environmental risks of enterprises and reward enterprises with good environmental performance through favorable cargo interest rates, Etc. However, there is a risk that companies may not follow through on their disclosures, i.e., they do not perform the environmental actions they have disclosed. Ingram [17] first found no significant correlation between environmental performance and environmental information disclosure, although several studies have since demonstrated the relationship between environmental performance and information disclosure. However, in practice, the situation of "inconsistent words and actions" in environmental information disclosure is still objective. This paper measures environmental behavior in terms of substantive environmental investment rather than environmental disclosure, which is a more direct and realistic response to enterprises’ fulfillment of environmental responsibility. Further, it explains its impact on the enterprises’ debt financing. Therefore, this paper proposes research hypothesis H1a:

H1a: The higher the investment in environmental protection by heavy pollution enterprises, the more new loans enterprises receive.

Debt financing of enterprises includes short-term debt financing and long-term debt financing. Short-term debt financing is mainly used for more liquid assets, and the structure and value of an enterprise’s assets are not likely to change significantly in a short period. Therefore, compared with long-term debt financing, short-term debt financing allows creditors to have timely information about the production and operation of the firm and facilitates creditors’ supervision and control [18,19]. Stiglitz and Weiss [20] show that when information asymmetry is very high, and the risk of default is high, firms cannot obtain long-term debt financing. Environmental issues are highly specialized and implicit, and there is a severe information asymmetry between creditors and lenders about the environmental performance of enterprises.

Moreover, according to Porter and Vander Linde’s [21] "Porter’s hypothesis", it is believed that while appropriate environmental regulation can increase the production costs of firms, it can also promote their technological innovation and productivity, which may further compensate the cost of environmental pollution control and bring tangible economic benefits. Economic benefits, and ultimately win a competitive advantage in the marketplace. In other words, an enterprise’s investment in environmental protection will increase production and operating costs in the short term. However, in the long term, its investment and improvement of green production equipment and production technology will help to improve the utilization of raw materials and energy resources and reduce pollution treatment costs, and from the outside of the enterprise can gain a significant competitive advantage in the increasingly environmentally-conscious consumer market, so in general, the long-term economic benefits are significant.

In summary, while good environmental performance has positive externalities, it may increase production costs within the company in the short term. The benefits of good environmental performance will be reflected in a long-term process. Based on this, this paper proposes research hypothesis H1b:

H1b: The higher the investment in environmental protection of pollution enterprises, the more new long-term loans enterprises receive.

### Green credit, environmental protection investment, and debt financing for heavy pollution enterprises

Monetary policy (credit policy) is an effective tool for the Chinese government to macro-regulate and govern firms. As a macroeconomic regulation tool, monetary policy is a vital tool to motivate firms to invest, and an easy monetary policy can promote expansionary investments by firms [22]. When firms face better investment opportunities, loose monetary policy will also improve the efficiency of firms’ capital allocation [23]. In addition, financial institutions will cooperate in implementing the national energy conservation and emission reduction strategy and give full play to their role in directing the flow of social capital and allocating credit resources. In February 2012, the CBRC issued the Green Credit Guidelines, a programmatic document for green credit business of financial institutions in China, which elevated green credit to a strategic level for the first time and promoted the integration of environmental and social responsibility into the assessment requirements for lending by banking financial institutions. This has increased credit support for energy conservation, environmental protection, and other green economic areas.

The establishment of the green credit audit system indicates that commercial banks have incorporated credit environmental risks into their daily risk management. Banks will pay more attention to the environmental performance of enterprises based on the implementation of green credit policies. The introduction of the green credit policy has made commercial banks take the environmental information disclosure of enterprises as an essential basis for judging the environmental risk of enterprises and assessing the risk of default, which makes the environmental performance of enterprises an essential factor affecting the external financing cost of enterprises. Companies with good environmental performance have relatively lower interest rates on their loans. In contrast, enterprises with poor environmental performance may be offered punitive off-rate loans.

Therefore, this paper proposes research hypothesis H2a and H2b.

H2a: The release of China’s Green Credit Guidelines in 2012 will enhance the positive relationship between environmental protection investment and new loans for heavy pollution enterprises.
H2b: The release of China’s Green Credit Guidelines in 2012 will enhance the positive relationship between environmental protection investment and new long-term loans of heavy pollution enterprises.

**Fig 2:**
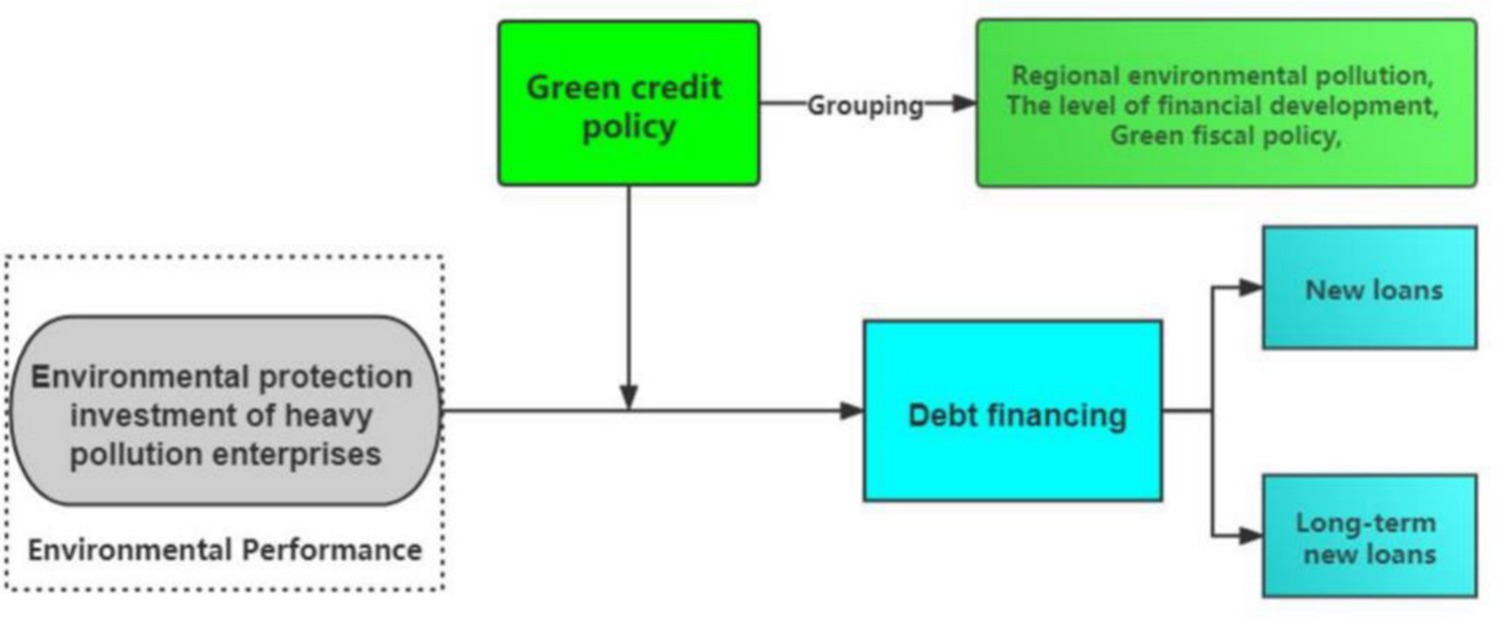
The structural framework.

To clarify this paper’s research questions and hypotheses, we provide a structural framework for the study in Fig 2.

## Research method

### Sample selection and data sources

The research samples selected in this paper are A-share listed companies in heavy pollution industries in Shanghai and Shenzhen stock markets from 2009 to 2017. According to the research needs, the sample screening process is as follows: excluding financial industry samples; Exclude ST company; After removing samples with seriously missing data, 5422 observation samples were finally obtained.

In this paper, the environmental protection investment data from the annual report of the enterprise note manual collection and processing, enterprise financial data mainly comes from Wind database and CSMAR database, regulating variable data through the China statistical yearbook, China’s financial, statistical yearbook, China industrial statistical yearbook, China city statistical yearbook and the provincial financial operation report received by hand. The Robust standard deviation of the multiple regression was modified, and the multiple regression was Cluster processed at the company level, and all continuous variables were Winsorized processed at the quantile of 1% and 99%.

### Definition of variables

The dependent variables in this paper are various indicators of debt financing capacity, explicitly including: interest-bearing debt financing (IBD), which refers to the increase of short-term borrowings of the current year, long-term borrowings due within one year, long-term borrowings, bonds payable, and long-term payables divided by the total assets at the end of the period; Short-term interest-bearing debt financing (SIBD) is the increase of the short-term borrowings of the current year and the long-term borrowings due within one year divided by the total ending assets; Long-term interest-bearing debt financing (LIBD) is the increase in the current year’s long-term borrowings, bonds payable and long-term payables divided by the total ending assets.

The independent variable in this paper is enterprise investment in environmental protection (EPI), mainly referring to Gi Li and Su Meng [24] and using the capital expenditure on environmental protection of the enterprise in the current year. Specifically, it is the capital expenditure on environmental protection of the current year divided by the total assets at the end of the period. Among them, the data of capital expenditure on environmental protection of enterprises are collected manually from the notes of "projects under construction" in the annual report of enterprises, mainly including energy conservation and environmental protection, sewage treatment, desulfurization and dust removal, waste gas treatment, low carbon, recovery, and recycling, Etc.

In order to study the influence mechanism of environmental protection investment of heavily polluting enterprises on their debt financing, the role of green credit plays. Adjustment variables were added: the date of issuance of the Green Credit Directive (POST). Specifically, the Green Credit Directive was issued in 2012 as the cut-off point and was defined as 1 after 2012 and 0 before 2012.

Enterprise debt financing will be affected by a variety of other factors, according to the Li and Liu [25] and Shen and Ma [26] study of the control variable regression to join this article: the company size (size), financial leverage (Lev) and profitability (roa), cash holdings (cash), equity financing ability (offer), age (age) and growth (growth), as shown in Table 2.

**Table 2:** Variable names and definitions.

Classification	Variable		Define
The dependent variable	IBD	Amount of interest-bearing debt financing	Short-term borrowings, long-term borrowings due within one year, long-term borrowings, bonds payable, and long-term payables/ending total assets
	SIBD	Amount of short-term interest-bearing debt financing	Short-term borrowings and long-term borrowings due within one year/ending total assets
	LIBD	Long-term interest-bearing debt financing amount	Long-term borrowings, bonds payable and long-term payables/ending total assets
The independent variables	EPI	Corporate investment in environmental protection	Total investment in environmental protection/total assets at the end of the period
Adjustment variable	post	Green credit start time	Greater than or equal to 1 in 2012;Less than 2012 is 0
Control variables	size	The enterprise scale	The natural log of total assets
	lev	Financial leverage	Total liabilities/ending total assets
	roa	profitability	Net profit/ending total assets
	cash	Cash holdings	Monetary capital/ending total assets
	offer	Equity financing capacity	Funds raised by rights offering or secondary issue at current period divided by total ending assets
	age	Enterprise age	Number of listed companies per year (year of observation—year of registration)
	growth	growth	(income from current year’s main business—income from last year’s main business)/income from last year’s main business

### Model construction

According to the assumptions and variables, Model 1 and Model 2 are established to test hypothesis 1 and hypothesis 2, respectively, as shown below:

$$Y_{i,t}=\alpha +\beta_{1}{EPI}_{i,t}+\sum Controls+\varepsilon$$ (1)

$$Y_{i,t}=\alpha +\beta_{1}{EPI}_{i,t}+\beta_{2}post+\beta_{3}{EPI}_{i,t}\times post+\sum Controls+\varepsilon$$ (2)

## Empirical results and analysis

### Descriptive statistics

As can be seen from the descriptive statistics in Table 3, the average value of short-term interest-bearing debt financing of enterprises is 0.1396, and that of long-term interest-bearing debt financing is 0.0677, indicating that heavy polluting enterprises are still more likely to use short-term debt for financing. The average value of the corporate investment in environmental protection is 0.0031, indicating a significant difference in corporate investment in environmental protection, and at least more than 50% of enterprises do not invest in environmental protection.

**Table 3:** Descriptive statistics.

Variables	N	Mean	Standard Deviation	Minimum	Median	Maximum
IBD	5422	0.2077	0.1646	-0.0124	0.1933	0.6378
SIBD	5422	0.1396	0.1249	-0.0242	0.1176	0.5256
LIBD	5422	0.0677	0.0908	-0.0177	0.0215	0.3972
EPI	5422	0.0031	0.0102	0	0	0.0697
post	5422	0.6865	0.4640	0	1	1
size	5422	21.9902	1.3135	19.16	21.8	25.91
lev	5422	0.4522	0.2258	0.0441	0.4476	1.0771
roa	5422	0.0352	0.0676	-0.2366	0.0322	0.2249
cash	5422	0.1642	0.1267	0.0076	0.1267	0.6094
offer	5422	0.0748	0.3025	0	0	2.4255
age	5422	14.9769	4.8762	4	15	27
growth	5422	0.1716	0.5532	-0.5738	0.0846	4.0949

### Correlation test

Table 4 below shows the correlation coefficients of the variables in this paper.

**Table 4:** Correlation coefficients.

	IBD	SIBD	LIBD	EPI	post	size	lev	roa	cash	offer	age	growth
IBD	1											
SIBD	0.831***	1										
LIBD	0.645***	0.119***	1									
EPI	0.111***	0.044***	0.136***	1								
post	-0.102***	-0.115***	-0.025*	0.030**	1							
size	0.418***	0.155***	0.543***	0.105***	0.130***	1						
lev	0.730***	0.629***	0.448***	0.068***	-0.095***	0.309***	1					
roa	-0.378***	-0.393***	-0.130***	-0.017	-0.022	0.041***	-0.458***	1				
cash	-0.405***	-0.314***	-0.302***	-0.064***	-0.049***	-0.234***	-0.425***	0.314***	1			
offer	-0.047***	-0.013	-0.071***	0.005	0.066***	-0.145***	0.010	-0.019	0.010	1		
age	0.062***	0.010	0.095***	0.033**	0.259***	0.107***	0.202***	-0.106***	-0.161***	0.046***	1	
growth	-0.016	-0.026*	0.010	-0.016	-0.068***	-0.004	0.012	0.199***	0.055***	0.060***	-0.012	1

Note: T-statistics in parentheses

***, ** and * indicate the significance at the level of 1%, 5% and 10%, respectively.

### Analysis of regression results

#### Main regression results

The regression results are shown in Table 5, in which columns 1–3 represent interest-bearing debt financing, long-term interest-bearing debt financing, and short-term interest-bearing debt financing, respectively, as can be seen from the data in the table. The estimated parameter value of explanatory variable EPI in column 1 is significantly positive at the 1% level, indicating that the environmental protection investment of heavily polluting enterprises, the more they will be able to increase their interest-bearing debt financing verifies Hypothesis 1a. When heavy polluting enterprises invest in environmental protection, they will establish a better "green image" and reduce the high risk caused by environmental uncertainty. Therefore, they can obtain financing convenience in the debt capital market. In particular, the parameter estimate of explanatory variable EPI in column 2 is significantly positive at the 1% level, while the parameter estimate in column 3 is not significant. It shows that the environmental protection investment of heavily polluting enterprises mainly increases its long-term interest-bearing debt financing, while the effect on short-term interest-bearing debt financing is not obvious, which verifies the research hypothesis 1b. Short-term debt financing of enterprises is usually used for assets with strong liquidity, so creditors of short-term debt have a relatively clear and comprehensive grasp of enterprises’ business situation and solvency. However, long-term debt’s more serious information asymmetry leads to a higher risk of default, so a good "green image" can better alleviate the information asymmetry between creditors and enterprises.

**Table 5:** Main regression results.

	(1)	(2)	(3)
	IBD	LIBD	SLBD
EPI	0.775***	0.543***	0.189
	(4.44)	(3.95)	(1.17)
size	0.030***	0.029***	0.001
	(8.32)	(16.47)	(0.37)
lev	0.422***	0.096***	0.324***
	(16.70)	(9.78)	(15.56)
roa	-0.262***	-0.004	-0.238***
	(-5.96)	(-0.18)	(-6.53)
cash	-0.110***	-0.057***	-0.059***
	(-4.49)	(-4.92)	(-2.87)
offer	-0.005	-0.005*	-0.001
	(-0.73)	(-1.82)	(-0.17)
age	-0.003***	-0.000	-0.003***
	(-4.75)	(-0.71)	(-4.85)
growth	0.000	-0.000	0.001
	(0.08)	(-0.14)	(0.22)
constant	-0.563***	-0.603***	0.043
	(-7.98)	(-16.31)	(0.77)
Ind	Yes	Yes	Yes
Year	Yes	Yes	Yes
N	5422	5422	5422
R^2^	0.605	0.444	0.459
Adjust R^2^	0.60	0.44	0.46

Note: T-statistics in parentheses

***, ** and * indicate the significance at the level of 1%, 5% and 10%, respectively.

### Green credit policy, environmental protection of heavy polluting enterprises and debt financing

The regression results are shown in Table 6. The parameter estimation of the interaction item POST ×EPI in the second column is positive and significant at the level of 1%, but it is also found that the parameter estimation in the third column is negative and significant at the level of 5%. According to the contents of the Green Credit Directive and previous studies, the policy has the effect of "punishment", that is, it will restrict some credit projects of enterprises that pollute the environment. Usually, heavily polluting enterprises will receive money as soon as possible using short-term financing to expand production and operation of enterprises further produce economic benefits, so the Banks in the credit approval stage of heavy pollution enterprise loan of the project such as this kind of violation of energy conservation and environmental protection policy to limit. This paper found that the green credit guidelines could suppress environmental protection investment’s positive impact on short-term debt financing, the negative significant regression results.

**Table 6:** The regression results of green credit policy impact.

	(1)	(2)	(3)
	IBD	LIBD	SLBD
EPI	0.708**	0.082	0.631**
	(2.31)	(0.40)	(2.15)
post	-0.035***	0.000	-0.035***
	(-2.91)	(0.05)	(-3.31)
post×EPI	0.095	0.653***	-0.626**
	(0.32)	(2.92)	(-2.21)
size	0.030***	0.029***	0.001
	(8.32)	(16.50)	(0.37)
lev	0.422***	0.096***	0.324***
	(16.70)	(9.76)	(15.57)
roa	-0.262***	-0.006	-0.236***
	(-5.97)	(-0.27)	(-6.48)
cash	-0.110***	-0.057***	-0.059***
	(-4.49)	(-4.90)	(-2.88)
offer	-0.005	-0.005*	-0.001
	(-0.74)	(-1.86)	(-0.15)
age	-0.003***	-0.000	-0.003***
	(-4.75)	(-0.75)	(-4.82)
growth	0.000	-0.000	0.001
	(0.08)	(-0.10)	(0.19)
constant	-0.563***	-0.601***	0.042
	(-7.97)	(-16.32)	(0.75)
Ind	Yes	Yes	Yes
Year	Yes	Yes	Yes
N	5422	5422	5422
R^2^	0.605	0.445	0.460
Adjust R^2^	0.60	0.44	0.46

Note: T-statistics in parentheses

***, ** and * indicate the significance at the level of 1%, 5% and 10%, respectively.

In addition to a more complete and detailed analysis from the view of policy content, found that the green credit guide also does not allow to ignore other roles, in particular, that can provide financial support for enterprise’s environmental protection and energy-saving project, give full play to the role of the green financial rational allocation of funds, guide the polluting enterprises in the direction of the sustainable development. The environmental protection projects of enterprises are usually characterized by significant initial investment, a long investment period, and slow recovery. Therefore, enterprises need more long-term capital support when making environmental protection investments. At this time, green credit will provide a long-term guarantee for these environmental protection projects. The regression results fully demonstrate the multi-functional and comprehensive policy role of the Green Credit Directive, which restricts short-term pollution projects of heavily polluting enterprises and supports long-term environmental protection and energy conservation projects. The final result is that the overall interest-bearing debt financing is not significant in the regression results.

After implementing the Green Credit Directive in 2012, the positive impact of environmental protection investment of heavily polluting enterprises on debt financing has been enhanced, which is mainly reflected in the impact on long-term debt financing. It shows that the Guidelines support policies to a certain extent, so Hypothesis 2b is verified.

Further, heavy polluting enterprises are divided into state-owned enterprises and non-state-owned enterprises according to the nature of their equity, large-scale enterprises, and small-scale enterprises according to the size of their enterprises. Then the impact of long-term debt financing is grouped, and regression is conducted. It can be seen from the regression data in Table 7 that the parameter estimation of the interaction item POST ×EPI is positive only in columns 2 and 4 and significant at the level of 1%. This indicates that after the release of the Green Credit Guidelines in 2012, the positive correlation between environmental protection investment of heavily polluting enterprises and new long-term debt is more significant. However, this effect only plays a significant role for state-owned enterprises and large-scale enterprises. It shows that green credit is a way of credit in essence as a macro means of government regulation. After the introduction of this new policy, banks still maintain a relatively conservative way of lending. Due to the nature, performance, and size of state-owned and large-scale enterprises, they have a more vital ability to resist risks, so banks are more inclined to lend to them.

**Table 7:** Grouped regression results of green credit policy impact for SOEs and non-SOEs, large and small-enterprises.

	(1)	(2)	(3)	(4)	(5)
	LIBD	SOE	non-SOE	Large-enterprises	Small-enterprises
EPI	0.082	0.039	0.042	-0.020	0.035
	(0.40)	(0.11)	(0.21)	(-0.06)	(0.24)
post	0.000	-0.020	0.035***	-0.022	0.048***
	(0.05)	(-1.62)	(2.76)	(-1.62)	(3.55)
post×EPI	0.653***	1.064***	0.296	0.891***	0.087
	(2.92)	(2.86)	(1.41)	(2.64)	(0.35)
size	0.029***	0.024***	0.030***	0.019***	0.025***
	(16.50)	(9.16)	(11.65)	(5.50)	(8.67)
lev	0.096***	0.134***	0.074***	0.189***	0.054***
	(9.76)	(7.64)	(8.24)	(9.58)	(7.43)
roa	-0.006	0.039	-0.027	0.043	-0.003
	(-0.27)	(0.98)	(-1.21)	(1.00)	(-0.14)
cash	-0.057***	-0.112***	-0.028***	-0.109***	-0.031***
	(-4.90)	(-4.48)	(-2.63)	(-4.03)	(-3.67)
offer	-0.005*	-0.010	-0.000	0.010	-0.002
	(-1.86)	(-1.58)	(-0.08)	(1.14)	(-0.75)
age	-0.000	-0.000	-0.000	-0.001	-0.000
	(-0.75)	(-0.21)	(-0.19)	(-1.15)	(-0.18)
growth	-0.000	-0.001	0.001	-0.003	0.001
	(-0.10)	(-0.35)	(0.53)	(-1.11)	(0.42)
constant	-0.601***	-0.504***	-0.621***	-0.404***	-0.496***
	(-16.32)	(-9.33)	(-11.33)	(-5.53)	(-8.34)
Ind	Yes	Yes	Yes	Yes	Yes
Year	Yes	Yes	Yes	Yes	Yes
N	5422	2425	2997	2708	2714
R^2^	0.445	0.446	0.357	0.413	0.240
Adjust R^2^	0.44	0.44	0.35	0.41	0.23

Note: T-statistics in parentheses

***, ** and * indicate the significance at the level of 1%, 5% and 10%, respectively.

### Robustness test

Replace the measure of the dependent variable. According to Li Zhijun and Wang Shanping [27], interest-bearing debt financing is replaced by new borrowing. Specifically, new borrowing is (long-term borrowing at the end of the year + short-term borrowing at the end of the year—long-term borrowing at the beginning of the year—short-term borrowing at the beginning of the year)/annual operating income. Replace long-term interest-bearing debt financing with new long-term borrowing, specifically (long-term borrowing at the end of the year—long-term borrowing at the beginning of the year)/annual operating revenue. The main regression analysis was re-conducted with the new dependent variable, and the results were consistent with the previous results.

They were lagging independent variables and control variables. This paper mainly studies that environmental protection investment of heavily polluting enterprises will promote their debt financing, but enterprises may have more funds to invest in environmental protection activities after obtaining more debt financing. In order to alleviate the endogeneity of mutual causation, the text firstly delayed both the explanatory variables and the control variables of the model for one period and then regressed, and the results were consistent with the previous analysis.

Add new control variables. After the financial crisis in 2008, China launched a series of economic policies to stimulate national economic development. In order to consider the potential impact of macroeconomic factors on enterprise investment and debt financing, the problem of missing variables is mitigated. Referencing Wenchao Ma and Yongjun Tang [28], Dongwei Su and Lili Lian [29], this paper added the control variables of GDP growth rate (G_GDP) and the proportion of the secondary industry (SIR) as the control variables, reestimated the models (1) and (2), and found that the empirical results were consistent with the previous analysis.

## Further analysis

This paper further analyzes the mechanism of the role of green credit policy from the perspectives of regional environmental pollution, the level of financial development, and green fiscal policy and focuses on the impact on long-term debt financing. All these factors affect enterprises’ environmental protection investment and further influence their debt financing, thus potentially affecting the regulating role of green credit policy in it.

### The impact of local environmental pollution

According to Ma Winchao and Tang Yongjun [28], the more serious the pollution level in the province to which the firm belongs, the greater the incentive for environmental constraints and environmental protection investment. Under the central government’s environmental regulation, the regional environmental regulation will face strategic competition, with provinces looking to regions with good environmental regulation [30]. The more serious the regional environmental pollution is, the more stimulating the competition between provinces will be, and the more stringent environmental pollution regulation will be. At this time, heavily polluting enterprises sensitive to environmental regulation will be under tremendous pressure from local governments. According to Porter’s [21] "innovation compensation effect", firms may have a positive response strategy to cope with local environmental regulatory pressure. Specifically, firms will respond to local environmental regulatory requirements by increasing the scale of environmental protection investment. In addition, from the perspective of enterprises, the actual marginal effect of investment in environmental protection is more pronounced for enterprises in areas with severe environmental pollution than for those with little environmental pollution, so the enthusiasm of enterprises to invest in environmental protection is also higher. Based on this, this paper introduces "regional industrial sulfur dioxide emissions" as a proxy variable to measure regional environmental pollution, divides the entire sample into high and low local environmental pollution levels according to the level of emissions, and performs group regressions.

The results of the group regressions are reported in Table 8. The results show that green credit and environmental investment are both significantly positive, and the results are more significant in the group with a high level of local environmental pollution. The regression results indicate that green credit policies have a more substantial effect in areas with high local environmental pollution levels than areas with low local environmental pollution levels. Moreover, when companies need to increase the scale of environmental protection investment, green credit can simultaneously respond to local environmental regulation and provide more funds for environmental improvement.

**Table 8:** Grouped regression results of the financial development impact.

	(1)	(2)	(3)
	All	The Level of Local Environmental Pollution is High	The Level of Local Environmental Pollution is Low
EPI	0.082	-0.035	0.197
	(0.40)	(-0.11)	(0.80)
post	0.000	-0.014	0.014
	(0.05)	(-1.06)	(1.04)
post×EPI	0.653***	0.779**	0.518*
	(2.92)	(2.43)	(1.80)
size	0.029***	0.031***	0.027***
	(16.50)	(11.74)	(12.28)
lev	0.096***	0.079***	0.112***
	(9.76)	(5.85)	(8.21)
roa	-0.006	-0.026	0.012
	(-0.27)	(-0.80)	(0.42)
cash	-0.057***	-0.078***	-0.035**
	(-4.90)	(-4.53)	(-2.37)
offer	-0.005*	-0.003	-0.006*
	(-1.86)	(-0.90)	(-1.72)
age	-0.000	0.000	-0.001
	(-0.75)	(0.32)	(-1.50)
growth	-0.000	0.004	-0.004
	(-0.10)	(1.36)	(-1.58)
constant	-0.601***	-0.641***	-0.565***
	(-16.32)	(-11.51)	(-12.28)
Ind	Yes	Yes	Yes
Year	Yes	Yes	Yes
N	5422	2658	2764
R^2^	0.445	0.435	0.463
Adjust R^2^	0.44	0.43	0.46

Note: T-statistics in parentheses

***, ** and * indicate the significance at the level of 1%, 5% and 10%, respectively.

### The impact of financial development

Xie Weimin, and Fang Hongxing [31] found the positive impact of regional financial development on enterprises’ R&D investment, and the development of credit markets can significantly promote enterprise innovation [32]. The main reason why financial development can promote firm innovation is that it makes capital available to firms. Moreover, in regions with developed financial markets, banks can obtain information about borrowers more timely and effectively, alleviating the information asymmetry issues. Love [33] found that financial development can improve the efficiency of resource allocation by reducing capital market imperfections caused by information asymmetry and contractual imperfections. Based on this, this paper uses Fan and Wang [34]’s study of China’s marketization index and introduces the "marketization of the financial sector" sub-index as a proxy variable to measure the level of financial development in each region, and divides the whole sample into high and low financial development according to the index level for group regressions.

The results of the group regressions are reported in Table 9. The results show that green credit and environmental investment are only significantly positive in the low financial development group, while positive but insignificant in those with high financial development. The regression results indicate that green credit is more substantial in areas with low financial development than areas with high financial development. This means that in areas with low financial development, information asymmetry is more severe, and banks cannot effectively obtain information about borrowing enterprises. At this time, green credit policy can instead play its role as a macroeconomic regulation policy instrument to allocate resources effectively. The heavily polluting enterprises in areas with weak financial development can play a complementary role in financing their environmental protection actions.

**Table 9:** Grouped regression results of the local environmental pollution impact.

		(1)		(2)		(3)
	All		The Level of Financial Development is High		The Level of Financial Development is Low	
EPI		0.082		0.349		-0.288
		(0.40)		(1.40)		(-0.99)
post		0.000		0.018		-0.015
		(0.05)		(1.39)		(-1.17)
post×EPI		0.653***		0.286		1.165***
		(2.92)		(1.05)		(3.47)
size		0.029***		0.028***		0.030***
		(16.50)		(10.38)		(12.99)
lev		0.096***		0.094***		0.096***
		(9.76)		(6.88)		(7.19)
roa		-0.006		-0.002		0.000
		(-0.27)		(-0.06)		(0.01)
cash		-0.057***		-0.025*		-0.090***
		(-4.90)		(-1.76)		(-5.00)
offer		-0.005*		-0.003		-0.007**
		(-1.86)		(-0.83)		(-2.03)
age		-0.000		-0.000		-0.000
		(-0.75)		(-0.48)		(-0.48)
growth		-0.000		-0.000		-0.000
		(-0.10)		(-0.11)		(-0.14)
constant		-0.601***		-0.590***		-0.600***
		(-16.32)		(-10.53)		(-12.26)
Ind		Yes		Yes		Yes
Year		Yes		Yes		Yes
N		5422		2656		2766
R^2^		0.445		0.426		0.448
Adjust R^2^		0.44		0.42		0.44

Note: T-statistics in parentheses

***, ** and * indicate the significance at the level of 1%, 5% and 10%, respectively.

### The impact of green fiscal policy

According to the study by Yuan Yijun and Kong Fanbin [35], the local fiscal expenditure on environmental protection falls into the category of public finance, which can lay a good foundation for enterprises to engage in subsequent pollution treatment or energy conservation and emission reduction and can reduce the market risk. It can be seen that the government’s financial expenditure on environmental protection can serve as an excellent example for enterprises to guide them to invest in environmental protection. Moreover, when the more the government spends on environmental protection, the more government subsidies enterprises may receive for their environmental protection actions, the more the enterprises’ environmental protection costs will be reduced. Ultimately, the benefits gained by enterprises outweigh the costs, and their motivation to invest in environmental protection will be enhanced, and they will be more active in environmental protection investment. This paper introduces government spending on energy conservation and environmental protection as a proxy variable for green fiscal policy. Precisely, government energy conservation and environmental protection expenditure is calculated as the ratio of the provincial energy conservation and environmental protection expenditure to the GDP of the province, and the entire sample is divided into high and low green fiscal policy by the amount of government energy conservation and environmental protection expenditure for group regressions.

The results of the group regressions are reported in Table 10. The results show that green credit and environmental investment are significantly positive only in the low green fiscal policy group, while positive but not significant in the high green fiscal policy group. The regression results indicate that green credit is more substantial in areas with low government environmental spending than high. This suggests that the green credit policy as a macroeconomic management tool in the capital market can play, to a certain degree, an "alternative" role compared to government control. When the government’s environmental spending is relatively lacking, enterprises can provide financial support to adopt environmental protection actions and increase their incentive to invest in environmental protection.

**Table 10:** Grouped regression results of the green fiscal policies impact.

	(1)	(2)	(3)
	All	The Level of Green Fiscal Policies is High	The Level of Green Fiscal Policies is Low
EPI	0.082	0.195	-0.055
	(0.40)	(0.57)	(-0.27)
post	0.000	-0.013	0.018
	(0.05)	(-0.99)	(1.53)
post×EPI	0.653***	0.520	0.839***
	(2.92)	(1.40)	(3.42)
size	0.029***	0.030***	0.027***
	(16.50)	(13.75)	(10.93)
lev	0.096***	0.084***	0.113***
	(9.76)	(7.75)	(7.53)
roa	-0.006	-0.002	-0.004
	(-0.27)	(-0.08)	(-0.13)
cash	-0.057***	-0.074***	-0.035**
	(-4.90)	(-5.10)	(-2.16)
offer	-0.005*	-0.007**	-0.004
	(-1.86)	(-2.06)	(-0.87)
age	-0.000	-0.000	-0.001
	(-0.75)	(-0.33)	(-1.22)
growth	-0.000	0.001	-0.001
	(-0.10)	(0.50)	(-0.49)
constant	-0.601***	-0.621***	-0.576***
	(-16.32)	(-12.96)	(-11.22)
Ind	Yes	Yes	Yes
Year	Yes	Yes	Yes
N	5422	2697	2725
R^2^	0.445	0.473	0.421
Adjust R^2^	0.44	0.47	0.42

Note: T-statistics in parentheses

***, ** and * indicate the significance at the level of 1%, 5% and 10%, respectively.

## Conclusions and implications

This paper investigates whether environmental investments by heavy pollution enterprises help their debt financing in the institutional background of China’s continued implementation of green credit policies. Based on the data of heavily polluting enterprises from 2009–2017, this paper finds that the higher the environmental protection investment of heavily polluting enterprises, the more new loans the enterprises can obtain, especially more new long-term loans in the institutional context of green credit policy, the importance of environmental protection investment of enterprises in obtaining new long-term borrowing increases. Further, the effects of green credit policy implementation are investigated by examining how regional environmental pollution, green fiscal policy, and the level of financial development affect the environmental investments of heavily polluting enterprises.

The findings of this paper have important implications for scholars, practitioners, and policymakers. First, this paper adds a new era of policy influences over traditional studies of corporate investment and debt financing, expanding the literature on the economic consequences of heavy pollution enterprises’ environmental investments, i.e., enhancing the availability of debt financing. This helps heavily polluting firms be more strategic in maintaining their sustainable development in response to China’s increasingly stringent environmental regulations. Second, this paper adds a study of green credit at the micro-level of firms, verifying that green credit policies can, to a certain extent, enhance the scale of firms’ environmental investments and further facilitate their access to debt financing, especially long-term interest-bearing debt financing. The study proves that green credit policies achieve policy financial support and resource allocation, which helps to understand the governance effects of green credit better. Finally, the paper expands more deeply about the implementation effect of green credit policy, and the study finds that green credit policy still has some limitations and does not fully play its role. The effect on short-term interest-bearing debt financing is not apparent, and the effect is only significant for long-term debt financing of state-owned enterprises and large-scale enterprises. Green projects are usually characterized by significant initial investment, a long investment period, and slow recovery, so non-SOEs or small-scale enterprises may need more financial support from the financial market. Therefore, when banks implement green credit policies, they should fully consider the evaluation criteria and management process of enterprise lending to further develop differentiated methods and indicators to promote the efficient implementation of green credit policies.

Developed countries are more environmentally conscious, and the development of green finance is earlier and more mature. In developing countries, economic development will go through a rough and intensive process. This crude economic development, with high consumption, wasteful resources, and environmental pollution, is the main reason and influencing factor that restricts developing countries’ sustainable economic development and ecological environment. China is currently in economic transition to sustainable development, and green credit has been developed in China for more than ten years since 2007. Although China is not the earliest country to develop green finance, the implementation of green credit policies in China is rapid and effective. A study on the development of green credit in China, reflecting on the current situation, problems, and the impact of green credit on economic growth, has important implications for developing green finance globally and provides a reference for sustainable economic development in developing countries.

There are some limitations in the current research of this paper. First, due to data availability, this paper only uses data from China to explore the impact of green credit policies. The applicability of the findings of this paper in other countries and regions needs to be further verified, and future research can expand the research sample to a global scale. Second, since green credit policy is an emerging market-based environmental regulation tool in China, the state does not currently force companies to publish their specific green credit data, leading to a lack of depth when conducting our research. In the future, with the continuous development of green finance, the policy will undoubtedly become more and more standardized and sound, and the disclosure of corporate information will become more and more adequate. The study can use detailed corporate micro panel data for a more comprehensive and deeper discussion.
